# Iron (II) Polypyridyl Complexes as Antiglioblastoma Agents to Overcome the Blood-Brain Barrier and Inhibit Cell Proliferation by Regulating p53 and 4E-BP1 Pathways

**DOI:** 10.3389/fphar.2019.00946

**Published:** 2019-09-03

**Authors:** Huili Zhu, Chengli Dai, Lizhen He, Anding Xu, Tianfeng Chen

**Affiliations:** ^1^Department of Neurology and Stroke Center, The First Affiliated Hospital, Jinan University, Guangzhou, China; ^2^The First Affiliated Hospital and the Department of Chemistry, Jinan University, Guangzhou, China

**Keywords:** glioblastomas, blood-brain barrier, 4E-BP1, iron (II) polypyridyl complex, metal complex

## Abstract

**Background and Purpose:** It is urgently required to develop promising candidates to permeate across blood-brain barrier (BBB) efficiently with simultaneous disrupting vasculogenic mimicry capability of gliomas. Previously, a series of iron (II) complexes were synthesized through a modified method. Hence, the aim of this study was to evaluate anticancer activity of Fe(PIP)_3_SO_4_ against glioma cancer cells.

**Methods:** Cytotoxic effects were determined *via* MTT assay, and IC_50_ values were utilized to evaluate the cytotoxicity. Cellular uptake of Fe(PIP)_3_SO_4_ between U87 and HEB cells was conducted by subtracting content of the complex remaining in the cell culture supernatants. Propidium Iodide (PI)-flow cytometric analysis was used to analyze cell cycle proportion of U87 cells treated with Fe(PIP)_3_SO_4_. The reactive oxygen species levels induced by Fe(PIP)_3_SO_4_ were measured by 2'-deoxycoformycin (DCF) probe; ABTS assay was utilized to examine the radical scavenge capacity of Fe(PIP)_3_SO_4_. To study the bind efficiency to thioredoxin reductase (TrxR), Fe(PIP)_3_SO_4_ was introduced into solution containing TrxR. To verify if Fe(PIP)_3_SO_4_ could penetrate BBB, HBMEC/U87 coculture as BBB model was established, and penetrating capability of Fe(PIP)_3_SO_4_ was tested. *In vitro* U87 tumor spheroids were formed to test the permeating ability of Fe(PIP)_3_SO_4_. Acute toxicity and biodistribution of Fe(PIP)_3_SO_4_ were tested on mice for 72 h. Protein profiles associated with U87 cells treated with Fe(PIP)_3_SO_4_ were determined by Western blotting analysis.

**Results:** Results showed that Fe(PIP)_3_SO_4_ could suppress cell proliferation by inducing G2/M phase cycle retardation and apoptotic pathways, which was related with expression of p53 and initiation factor 4E binding protein 1. In addition, Fe complex could suppress cell proliferation by downregulating reactive oxygen species levels *via* scavenging free radicals and interaction with TrxR. Furthermore, Fe(PIP)_3_SO_4_ could permeate across BBB and simultaneously inhibited the vasculogenic mimicry-channel of U87 cells, suggesting favorable antiglioblastoma efficacy. Acute toxicity manifested lower degree of the complex compared with cisplatin and temozolomide.

**Conclusion:** Fe(PIP)_3_SO_4_ exhibited favorable anticancer activity against glioma cells associated with p53 and 4E binding protein 1, accompanied with negligible toxic effects on normal tissues. Herein, Fe(PIP)_3_SO_4_ could be developed as a promising metal-based chemotherapeutic agent to overcome BBB and antagonize glioblastomas.

## Introduction

**Figure 1:**
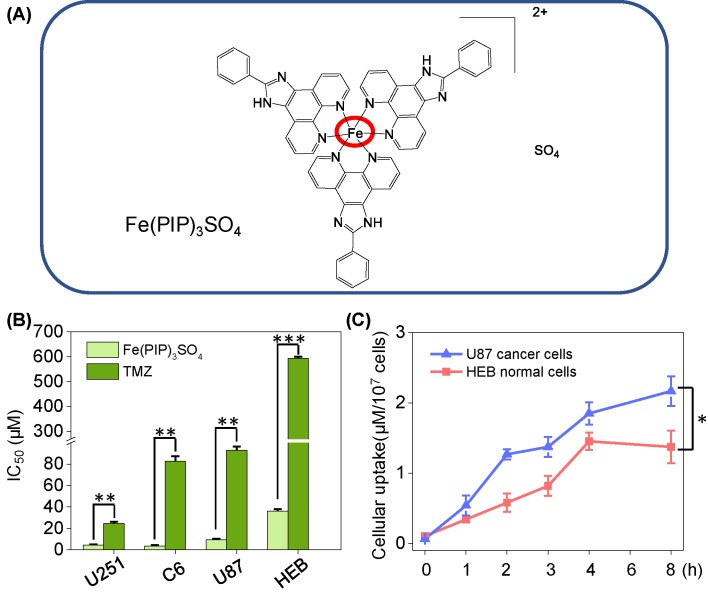
**(A)** Structure of iron (II) complex, Fe(PIP)_3_SO_4_. **(B)** IC_50_ values of Fe(PIP)_3_SO_4_ toward glioma cell lines (U87, U251, and C6 cells) and normal cell lines (HEB and CHEM5 cells) after incubation for 72 h. **(C)** Cellular uptake of Fe(PIP)_3_SO_4_ in U87 and HEB cells in 8-h treatment. Cells mentioned were incubated with Fe(PIP)_3_SO_4_ (10 μM) for consecutive time. Values are presented as means ± SD in triplicate. Significant difference between the treatment and control groups is indicated at the *P*<0.05 (*), *P*<0.01 (**), or *P*<0.001 (***) level.

Recently, researchers have investigated widely about metal-based complexes for cancer therapy, with various types of metallic antitumor candidates being developed for decades, such as gold (Au) and Au-NHCs complexes (Fung et al., 2017; Porchia et al., 2018; Williams et al., 2018), ruthenium (Ru) complexes (Tan et al., 2010), iridium (Ir) complexes (Cao et al., 2013), iron (Fe) complexes (Gou et al., 2016), etc. For instance, Che et al. found that Au (III) complexes containing special ligand could act as smart fluorescent probes and anticancer agents (Zou et al., 2013). Meanwhile, Ru polypyridyl complexes are suggested to act as thioredoxin reductase (TrxR)–targeted agents as promising anticancer candidates (Luo et al., 2014). Hence, Fe polypyridine derivates with lipophilicity in previous study were chosen for further study in antiglioma therapy (**Figure 1A**).

Glioblastomas have been considered as one of the lethal types of human primary brain tumors, which is characterized by diffusing invasion of distant brain tissue to a myriad of migrating cells and reduction in rate of cell apoptosis, consequently generating resistance to proapoptotic drugs (Kast et al., 2013). More than that, communication with and manipulation of other cells in the brain environs are associated with glioblastoma growth, leading to resistance to therapy (Broekman et al., 2018). The invasive glioma cells are capable to permeate across blood-brain barrier (BBB), followed by migrating into the brain, simultaneously impeding a majority of therapeutic cargos to get into the brain (Kijima et al., 2012; Harder et al., 2018). Besides, the vasculogenic mimicry (VM) capacity of gliomas has also been considered as an obstacle to get over the disease (Qiao et al., 2015).

In terms of therapies for glioblastomas, eukaryotic initiation factor 4E (eIF4E)–binding proteins (4E-BP1) is a downstream peptide in mammalian target of rapamycin (mTOR) critical pathway, which is a crucial regulator for cancer cells (Wolfson and Sabatini., 2017; Song et al., 2019; Woodcock et al., 2019), and belongs to a family of translation repressor proteins (Rojo et al., 2007; She et al., 2010). Nonphosphorylated and low-phosphorylated types of 4E-BP1 have been considered as active forms of 4E-BP1 and could bind to eIF4E, then inhibiting translation process of 5’ cap-dependent proteins and regulating protein synthesis (Jhanwar-Uniyal et al., 2015; Lin et al., 2018). One more to mention, DNA-alkylating temozolomide (TMZ), as an alkylating agent *via* oral chemotherapy, could effectively transit across BBB and inhibit cancer proliferation through interfering replication and repair of intracellular DNA damage (Stupp et al., 2002; Hart et al., 2013). Nevertheless, the side effects of TMZ in clinical applications have become apparent throughout years, including serious bone marrow toxicity (Kourelis et al., 2015), alopecia (Virmani et al., 2015), and local or systemic rash (Birsen et al., 2018), etc. These limitations noted in the therapeutic strategies of TMZ have prompted extensive and further researches about alternative and promising antiglioblastoma candidates.

To date, Fe (II) polypyridyl complexes have been considered as promising antiproliferative agents against cancer cells, including ferrocenyl-containing complexes (Zhao et al., 2012) and Fe (II/III) polypyridyl complexes (Gou et al., 2016; Xie et al., 2017). Herein, based on previous study, we studied the anticancer activities of Fe(PIP)_3_SO_4_ (Chen et al., 2015) against glioma U87 cells, including regulating the expression levels of p53 and 4E-BP1, triggering cell apoptosis and cycle arrest to inhibit cell proliferation, downregulating cellular reactive oxygen species (ROS) levels and TrxR activity, and penetrating across BBB, which was accompanied with negligible toxicity on normal tissues *in vivo*. Findings involved in this study will enhance our understanding of the biomedical applications of next-generation metal-based anticancer candidates.

## Materials and Methods

### Chemicals and Reagents

2′,7′-Dichlorodi-hydrofluorescein diacetate (DCFH-DA) probe, propidium iodide (PI), 3-(4,5-dimethyl-2-thiazolyl)-2,5-diphenyl-2-H-tetrazolium bromide (MTT), and BCA assay kit were purchased from Sigma-Aldrich (USA). The TrxR Activity Kit was brought from Cayman Chemical (Michigan, USA). The Annexin V Alexa Fluor 647/PI Apoptosis Detection Kit was purchased from Solarbio (Beijing, China). All the antibodies used in this study were obtained from Cell Signaling Technology (Beverly, MA, USA). Fe(PIP)_3_SO_4_ (PIP = 2-phenylimidazo[4,5-*f*] [1,10] phenanthroline) and Fe(IP)_3_SO_4_ (IP = imidazole[4,5-*f*] [1,10] phenanthroline) were synthesized in our laboratory according to previous report. In brief, mentioned 2 Fe complexes were prepared through Fe(NH_4_)_2_(-SO_4_)_2_·6H2O by drop, added with diamine ligands (ip, 0.33g; pip, 0.47 g). Electrospray ion source-mass spectrometry (ESI-MS) elemental analysis, ultraviolet visible light (UV-Vis), ^1^H-nuclear magnetic resonance (NMR), and ^13^C-nuclear magnetic resonance (NMR) were utilized for characterization (Chen et al., 2015). In addition, Fe(PIP)_3_SO_4_ and Fe(IP)_3_SO_4_ were dissolved in dimethyl sulfoxide.

### Cell Culture and Cytotoxic Effects

All the cancer and normal cell lines mentioned in this study, including mouse glioma cells (C6), human glioma cells (U87 and U251), normal human glial cells (HEB), and normal mouse glial cells (CHEM5), were purchased from American Type Culture Collection (Manassas, VA, USA). Cell lines were cultured in Dulbecco modified eagle medium (DMEM) supplemented with fetal bovine serum (10%), penicillin (100 U/ml), and streptomycin (50 U/ml) at 37 °C in a CO_2_ incubator (95% relative humidity, 5% CO_2_). Cytotoxic effects were determined by MTT assay. Briefly, IC_50_ values (half inhibitory concentrations) were determined by measuring cells’ ability to transform MTT to the purple formazan dye. The color intensity of the formazan solution was measured at 570 nm of wavelength by using a microplate spectrophotometer (Spectro Amax 250, USA).

### *In Vitro* Cellular Uptake of Fe(PIP)_3_SO_4_

Quantitative analysis of cellular uptake for Fe(PIP)_3_SO_4_ toward cancer and normal cells was conducted as previously described (He et al., 2015). Briefly, U87 cells were seeded into 6-well plates (2 × 10^5^ cells/ml) and then exposed to Fe(PIP)_3_SO_4_ (20 μM) for 1, 2, 3, 4, 6, and 8 h. Ultraviolet visible light spectrophotometer was used to measure UV-Vis absorption of Fe(PIP)_3_SO_4_ at a wavelength of 535 nm for estimated time points. Cellular uptake analysis was determined by subtracting the concentrations of complexes remaining in the cell culture supernatants, and this experiment was conducted on a microplate spectrophotometer (Spectro Amax 250, USA).

### Flow Cytometry Analysis

Flow cytometry was utilized to analyze cell cycle proportion of U87 cells in each stage of cycle proportion after well exposure to Fe(PIP)_3_SO_4_, and then this assay was conducted on a flow cytometer (CytoFLEX 5, USA). The experiment was conducted by using PI-flow cytometric analysis as previously described (Lai et al., 2015). Briefly, U87 cells (2 × 10^4^ cells/ml) were exposed to Fe(PIP)_3_SO_4_ (5, 10, 20, and 40 μM) for 36 h and 20 μM of concentration for 24, 36, 48, and 72 h. The treated cells were collected, fixed with 70% ethanol overnight, and then stained with PI in darkness for 2 h. For each experiment, 10,000 events per sample were recorded.

### Measurement of Intracellular ROS Generation

Intracellular ROS levels in U87 cells (2 × 10^5^ cells/ml) induced by Fe(PIP)_3_SO_4_ were measured by using a DCFH-DA probe as previously described (Xie et al., 2014). The relative DCF fluorescence intensity of the treated cells was expressed as the percentage of control (% of control), which represents the relative ROS levels induced by Fe(PIP)_3_SO_4_ in U87 cells; excitation and emission wavelengths for DCF were 488 and 525 nm, respectively. This assay was conducted on a microplate reader (Spectra Max M5, MD, USA).

### ABTS+ Assay

First, ABTS solution was prepared with the characteristic absorbance at 734 nm as previously described (Liu et al., 2017). Then, 20 μl of Fe(PIP)_3_SO_4_ was added to a 96-well plate at different final concentrations, and 100 μl of ABTS solution (300 μM) was added to each well containing Fe(PIP)_3_SO_4_. Besides, ABTS solution treated with phosphate-buffered saline (PBS) was introduced as control group. Radical scavenging capability of Fe(PIP)_3_SO_4_ was calculated as the percentage of the absorption intensity of the negative control group. The absorbance value was measured at 734 nm using a microplate spectrophotometer (Spectro Amax 250, USA).

### Binding of Fe(PIP)_3_SO_4_ to a TrxR

In this experiment, Fe(PIP)_3_SO_4_ (4 μM) was introduced into a system containing TrxR peptide at a ratio of 1:1 (v/v). Ultraviolet visible light spectra and high-performance liquid chromatography (HPLC) analysis were used to determine the binding efficiency between Fe(PIP)_3_SO_4_ and TrxR after mixing for 12 h. Ultraviolet visible light spectra analysis was carried out on an ultraviolet spectrophotometer (UH4150; Hitachi, Japan).

### High-Performance Liquid Chromatography Analysis

Fe(PIP)_3_SO_4_ in methanol (HPLC grade) was injected into an Agilent C18 column (250 × 4.6 mm), previously equilibrated with solution that consists solvent A (0.4% aqueous phosphoric acid, v/v) and solvent B (methanol). Compounds were eluted from the column according to the following program: 50% A in 0 to 30 min, 0% A in 30 to 40 min. Flow rate was 1.0 ml/min, and the elute was monitored at 300 nm to acquire the chromatograms. High-performance liquid chromatography analysis was performed by a 1260 Infinity II chromatography system (Agilent Technologies, USA).

### Determination of TrxR Inhibition

Pure proteins were extracted from U87 cells by cell lysis buffer, and then the protein concentrations were determined *via* the BCA assay. The inhibition effect of TrxR activity in U87 cells triggered by Fe(PIP)_3_SO_4_ at different concentrations within 50 min was determined using a TrxR assay kit (Cayman) according to the manufacturer’s instructions.

### Transportation Across the BBB of Fe(PIP)_3_SO_4_

To ascertain the permeability of Fe(PIP)_3_SO_4_ across BBB, HBMEC/U87 coculture as BBB mimic model was established according to previous report (Ying et al., 2010). Briefly, HBMEC cells (2 × 10^5^ cells/ml) were seeded in the upper chambers; U87 cells (2 × 10^4^ cells/ml) were seeded in the basolateral compartment of inset. The model was successfully prepared until the electric resistance of HBMEC on the upper chamber was charged over 250 Ω cm^2^. Additionally, transmittance ratio of Fe(PIP)_3_SO_4_ was measured by an UV spectrophotometer (Spectra Max M5, Maryland) at 535 nm as previously reported (Mo et al., 2016). Simultaneously, cellular uptake of Fe(PIP)_3_SO_4_ in U87 cells was measured by using a cell imaging multimode reader (Cytation 5, Bio Tek, USA).

### Destruction of Brain Cancer VM Channels Induced by Fe(PIP)_3_SO_4_

A Matrigel-based tube formation assay was used to illustrate the inhibition of VM channels of U87 cells. First, 100 μl of microthermal Matrigel was added into a prechilled plate and then allowed to solidify at 37 °C for 30 min. After that, U87 cells were incubated with Fe(PIP)_3_SO_4_ (2.5, 5, and 10 μM, respectively) and seeded into the Matrigel-coated wells. After 36-h incubation, the tubules were detected and photographed under an inverted microscope (IX51, Olympus, Japan).

### Penetrating Ability and Inhibitory Effects of Fe(PIP)_3_SO_4_ in Brain Tumor Spheroids

U87 tumor spheroids were established as previously described (Li et al., 2014). For spheroid formation, U87 cells (2 × 10^5^ cells/ml) were seeded in an ultralow attachment plate (Corning, USA) in DMEM medium. Then, the tumor spheroids (500 μl) were picked into a 6-well plate coated with 2% agarose (m/v) and incubated with Fe(PIP)_3_SO_4_ for 12 h. To determine the penetration ability of Fe(PIP)_3_SO_4_, tumor spheroids were first rinsed with PBS and then scanned by consecutive layers from top to middle zone *via* confocal laser scanning fluorescent microscope (Carl Zeiss, Germany). The bulk of U87 tumor spheroids was calculated using a fluorescence microscope (EVOS^®^ FL, Life Technologies, USA) in white light at different time points (0, 1, 2, 3, and 5 days), the formula *V* = (π × *d*_max_ × *d*_min_)/6, R = (*V*_i_/*V*_0_) ×100%, as previously reported (Mo et al., 2016).

### Western Blotting Analysis

Protein expression profiles associated with different signaling pathways in U87 cells (1 × 10^5^ cells/ml) treated with Fe(PIP)_3_SO_4_ (20 μM) were determined by Western blotting analysis (Chen and Wong, 2008). Total cellular protein of U87 cells treated with Fe(PIP)_3_SO_4_ was extracted by lysis buffer (Beyotime, China), the concentrations of proteins were determined by BCA assay. Expression levels of β-actin were used as internal standard to analyze the content of protein in each lane.

### Modified Annexin V-fluoresceine isothiocyanate/Propidium Iodide (FITC/PI) Apoptosis Assay

First, U87 cells (1 × 10^5^ cells/ml) were seeded in 10-cm dishes for 24 h to static adherence, and then Fe(PIP)_3_SO_4_ (5, 10, 20, and 40 μM, respectively) was introduced into each culture dish. After incubation for 36 h, U87 cells (including the floating cells in the supernant) were harvested and resuspended in PBS (with no calcium). To be stained efficiently, cells were washed with PBS first, then centrifuged followed by decanting the supernatant, and finally resuspended with PBS again (1 × 10^6^ cells/ml, 500 μl). The resuspended cells were mixed with binding buffer (50 μl) and Annexin V–fluoresceine isothiocyanate (FITC) (2.5 μl) +PI (2.5 μl), which was conducted in darkness at 25 °C for 15 min. This experiment was carried out on Epics XL-MCL flow cytometer (Beckman Coulter, Miami, FL). For each experiment, 10,000 events per sample were recorded.

### *In Vivo* Drug Accumulation in Brain Analysis

BALB/C mice (5 weeks, 18-22 g, no. 44007200055536) mentioned in this assay were obtained from the medical laboratory animal center in Guangdong Province. Briefly, mice were treated with Fe(PIP)_3_SO_4_ (4 and 8 mg/kg, respectively) through intravenous (i.v.) administration. At 24-, 48-, and 72-h time points, 3 mice in each group (n = 9, per group) were under euthanasia, and the brains were obtained. Quantitative analysis of Fe content in the brains was determined by inductively coupled plasma optical emission spectrometry (ICP-OES) analysis. This animal study was carried out in accordance with the principles of the Basel Declaration and recommendations of the Institutional Animal Use and Care regulations of Jinan University.

### Acute Toxicity Experiments

National Institutes of Health (NIH) mice (15-20 g, 28-42 days, no. 44007200058239) used in this study were provided by medical laboratory animal center in Guangdong Province. The mice were raised in a room (SPF grade, no. 00204639) with controlled temperature (20-25 °C) and appropriate humidity (40%-70%). Briefly, mice (n = 8, per group) were treated with Fe(PIP)_3_SO_4_, cisplatin, TMZ (4 mg/kg), and saline (i.v.). After 72 h, all mice groups were under euthanasia, and the heart, liver, spleen, lung, kidney, and brain tissues were taken out for detection of Fe content and hematoxylin-eosin (H&E) staining. The blood samples were separated from mice for hematological analysis at Guangzhou Overseas Chinese Hospital. All animal procedures were carried out in accordance with the principles of the Basel Declaration and recommendations of the Institutional Animal Use and Care regulations of Jinan University.

### Statistics Analysis

All data are expressed as mean ± standard deviation. Differences between the control and the experimental groups were analyzed using a 2-tailed Student *t* test. One-way analysis of variance was used for multiple group comparisons. Statistical analysis was performed using the SPSS statistical program version 13 (SPSS Inc., Chicago, IL, USA). Differences with *P* < 0.05 (*), *P* < 0.01 (**), or *P* < 0.001 (***) were considered statistically significant.

## Results

### Cytotoxic Effects and Cellular Uptake of Fe(PIP)_3_SO_4_

Cytotoxicity of Fe(PIP)_3_SO_4_ toward glioma cells (C6, U87 and U251 cells), normal human glial cells (HEB cells), and normal mouse glial cells (CHEM5 cells) was determined by MTT assay, compared with TMZ. IC_50_ values (half inhibition concentrations) were calculated to determine the cytotoxic effects of Fe(PIP)_3_SO_4_ on cancer and normal cell lines. As shown in **Figures 1A, B** and **Table S1**, IC_50_ values of Fe(PIP)_3_SO_4_ toward U87, U251, and C6 cells were found at 9.35, 4.44, and 3.54 μM, respectively, whereas those for TMZ were 92.87, 24.49, and 82.69 μM, respectively. It is interesting that the cytotoxic effect of Fe(PIP)_3_SO_4_ toward human normal cells was 3.88-fold lower than that of cancer cells, indicating its safety potency in future application. IC_50_ values of Fe(PIP)_3_SO_4_ and TMZ toward HEB cells (human normal glial cells) were 36.3 and 592.8 μM, respectively. Furthermore, cellular uptake of Fe(PIP)_3_SO_4_ toward U87 cells and HEB cells was further tested for 8-h incubation to examine the difference of cellular uptake between cancer and normal cell lines. **Figure 1C** shows that uptake content of Fe(PIP)_3_SO_4_ (10 μM) in U87 cells gradually rose up to 2.17 μM/10^7^ cells of Fe content at 8 h, and that of Fe(PIP)_3_SO_4_ (10 μM) toward HEB cells also showed moderate uptake increasement to 1.37 μM/10^7^ cells of Fe content during 8-h treatment.

### Cell Apoptosis and Cycle Arrest Induced by Fe(PIP)_3_SO_4_

**Figure 2:**
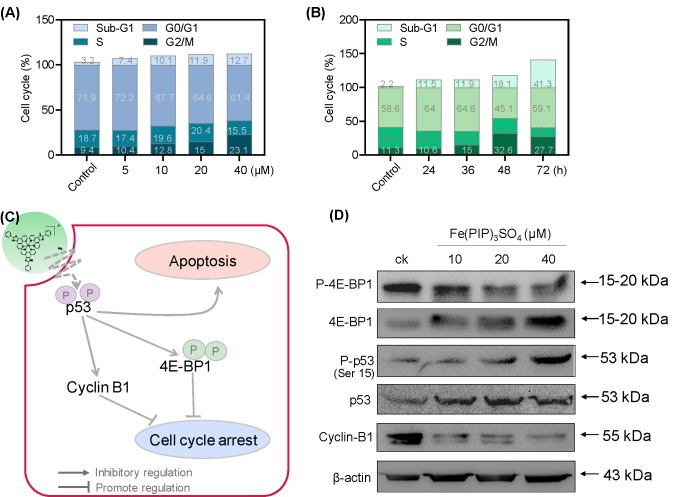
G2/M phase arrest and apoptotic cell death in U87 cells triggered by Fe(PIP)_3_SO_4_. Cell cycle analysis of G2/M phase arrest and apoptosis induced by Fe(PIP)_3_SO_4_ in U87 cells. Cells were exposed to **(A)** Fe(PIP)_3_SO_4_ (5, 10, 20, and 40 μM, for 36 h) and **(B)** Fe(PIP)_3_SO_4_ (20 μM for 24, 36, 48, and 72 h). **(C)** Schematic diagram of anticancer mechanism for Fe(PIP)_3_SO_4_ in U87 cells. **(D)** Effects of Fe(PIP)_3_SO_4_ on the expression levels of G2/M phase arrest and apoptotic cell death in cancer cells (treatment time 24 h).

**Figure 3:**
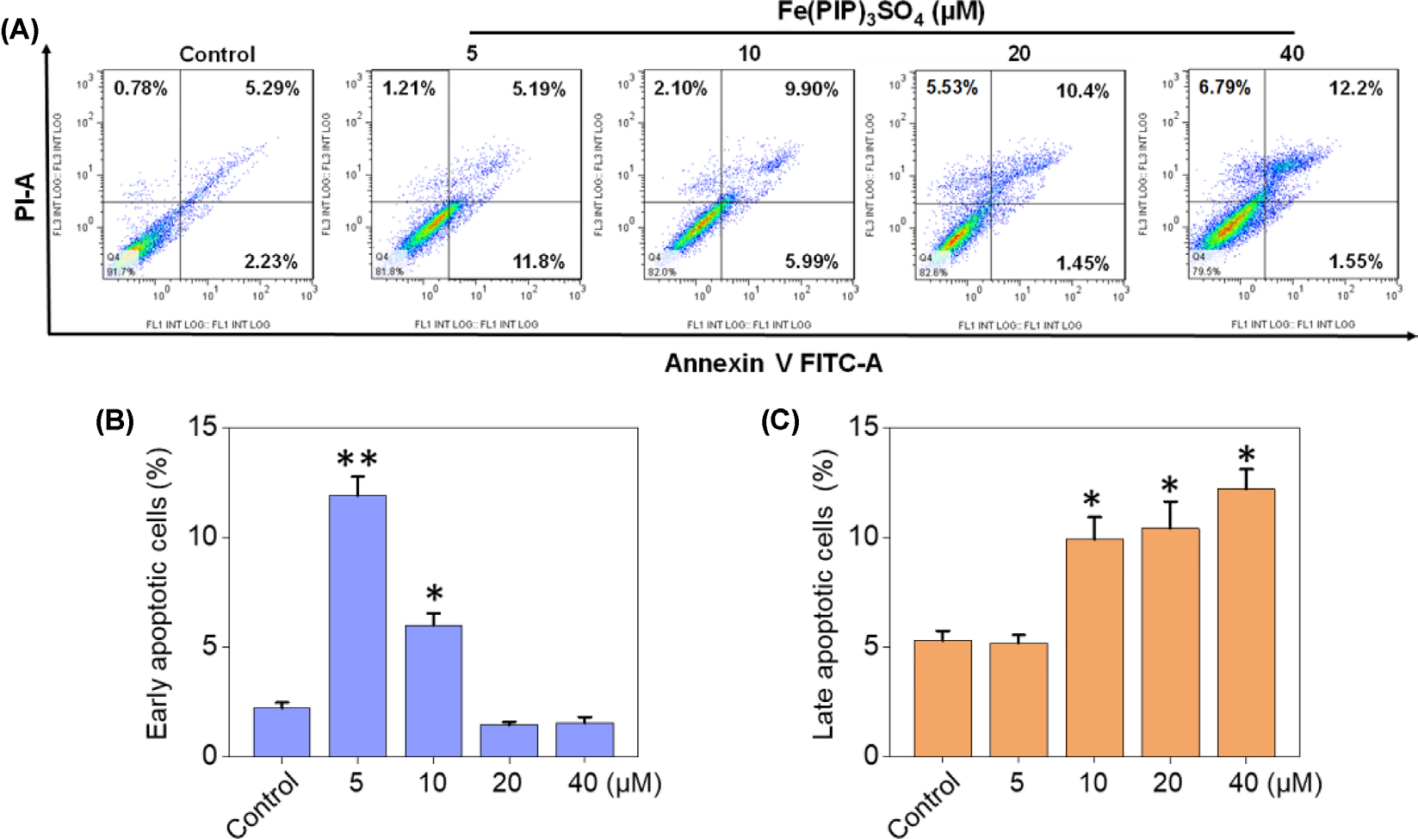
Cell apoptotic analysis induced by Fe(PIP)_3_SO_4_. **(A)** Flow cytometric analysis for Annexin V–FITC/PI in U87 cells treated with Fe(PIP)_3_SO_4_ (5, 10, 20, and 40 μM, respectively) for 36 h. The percentage of Annexin V–positive and PI-negative cells (bottom right) represents the proportion of early apoptotic cells; the percentage of Annexin V–positive and PI-positive cells (top right) represents the proportion of late apoptotic cells. **(B)** Quantitative analysis of early apoptotic U87 cells induced by Fe(PIP)_3_SO_4_ (5, 10, 20, and 40 μM, respectively) after 36 h of incubation. Values are presented as means ± SD in triplicate. Significant difference between the treatment and control groups was indicated at the *P*<0.05 (*) or *P*<0.01 (**) level. **(C)** Quantitative analysis of late apoptotic U87 cells induced by Fe(PIP)_3_SO_4_ (5, 10, 20, and 40 μM, respectively) after 36 h of incubation. Values are presented as means ± SD in triplicate, and significant difference between the treatment and control groups is indicated at the *P*<0.05 (*) level.

PI-flow cytometric analysis was conducted to investigate the mechanisms associated with cell death triggered by Fe(PIP)_3_SO_4_. U87 cells were incubated with Fe(PIP)_3_SO_4_ (5, 10, 20, and 40 μM, respectively) for 36 h, exhibiting gradual accumulation in the proportion of cells in G2/M phase, rising from 9.3% (control) to 23.1% (40 μM). Another overt augment could be noted in the subdiploid peak ascending from 3.2% (control) to 12.7% (40 μM). In addition, subdiploid peak markedly increased from 2.2% (control) to 41.3% (72 h) as the exposure time of Fe(PIP)_3_SO_4_ (20 μM) on U87 cells prolonged to 72 h, accompanied with slight increments in the G2/M phase proportion (**Figures 2A, B** and **Figure S1**), whereas no obvious proportion changes could be noticed in G0/G1 or S phase. **Figure 2C and D** showed that Fe(PIP)_3_SO_4_ could suppress expression of key signaling proteins, including cyclin B1 and p4E-BP1 involved in G2/M cell cycle arrest. Furthermore, regulation of p53 in U87 cells was also investigated after incubation with Fe(PIP)_3_SO_4_ (10, 20, and 40 μM, respectively) for 24 h. Moreover, Fe(PIP)_3_SO_4_ led to marked enhancement of p53 phosphorylation (Ser 15). Based on these findings, flow cytometric analysis of Annexin V–FITC/PI in U87 cells was utilized for further apoptotic analysis; results demonstrated that the percentage of Annexin V–positive and PI-negative cells (bottom right) was indicated for the proportion of early apoptotic cells, increasing from 2.23% (control) to 11.8% (5 μM) and 5.99% (10 μM), followed by descending to 1.45% and 1.55% (20 and 40 μM). The percentage of Annexin V–positive and PI-positive cells (top right) represents the proportion of late apoptotic cells, ascending from 5.29% (control) to 9.90% (10 μM), 10.4% (20 μM), and 12.2% (40 μM), respectively (**Figures 3A–C**).

### Downregulation of Intracellular ROS Generation by Fe(PIP)_3_SO_4_

**Figure 4:**
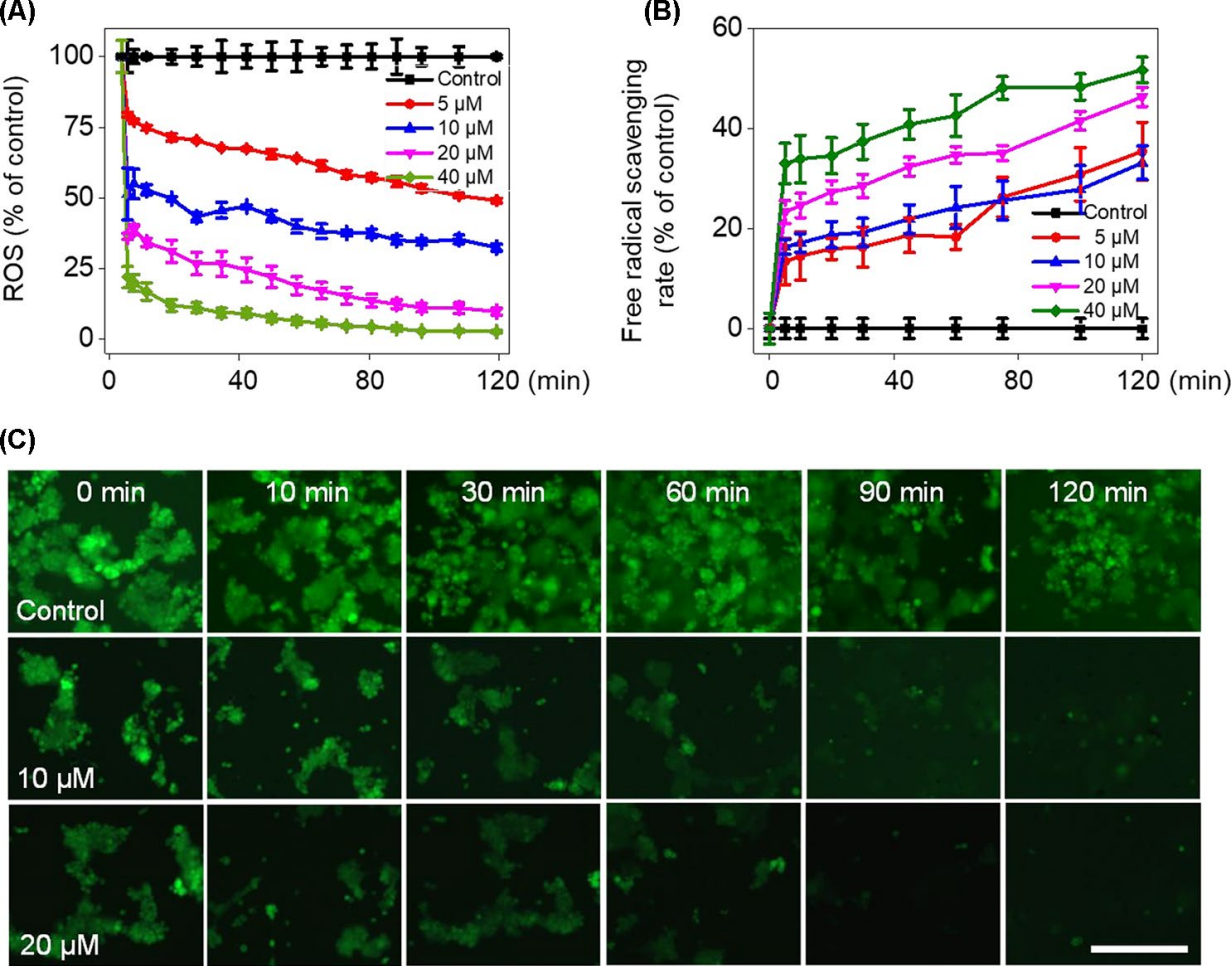
Downregulation of intracellular ROS levels of U87 cells induced by Fe(PIP)_3_SO_4_. **(A)** Intracellular ROS generation in U87 cells after treatment with Fe(PIP)_3_SO_4_ (5, 10, 20, and 40 μM, respectively) for 120 min. **(B)** The free radical–scavenging capacity of Fe(PIP)_3_SO_4_ (5, 10, 20, and 40 μM, respectively) in 120 min was determined by ABTS assay. Values are presented as means ± SD in triplicate. **(C)** The detection of ROS in U87 cell exposure to Fe(PIP)_3_SO_4_ in 120 min by fluorescence microscopy, as detected by DCF staining. Scale bar = 200 μm.

To verify whether Fe(PIP)_3_SO_4_ could trigger ROS-mediated cell death, DCFH-DA fluorescence assay was conducted to investigate ROS levels in U87 cells after incubation with Fe(PIP)_3_SO_4_ (5, 10, 20, and 40 μM, respectively). As shown in **Figure 4A**, ROS levels in U87 cells descended sharply after 10-min treatment (almost declining to 23% with 40 μM of Fe(PIP)_3_SO_4_), followed by moderate reduction nearly down to 13.5% in 120 min. To further study this phenomenon, fluorescence microscopy imaging was used to visualize the ROS downregulation process in U87 cells. As shown in **Figure 4C**, the fluorescent intensity of DCF probe in U87 cells was attenuated at 10 min induced by Fe(PIP)_3_SO_4_ (10 and 20 μM, respectively); fluorescent intensity gradually fainted as the exposure time extended to 120 min. Additionally, *in vitro* free radical–scavenging ability of Fe(PIP)_3_SO_4_ was examined by using an 2,2′-azino-bis (3-ethylbenzothiazoline-6-sulfonic acid) (ABTS^+^) scavenging assay. Results showed that the scavenge ability of Fe(PIP)_3_SO_4_ to free radicals gradually increased to 46% and 51.5% (20 and 40 μM) at 120 min (**Figure 4B**). Fe(PIP)_3_SO_4_ to free radicals gradually increased to 46% and 51.5% (20 and 40 μM) at 120 min (**Figure 4B**).

### Inhibition of Intracellular TrxR Induced by Fe(PIP)_3_SO_4_

**Figure 5:**
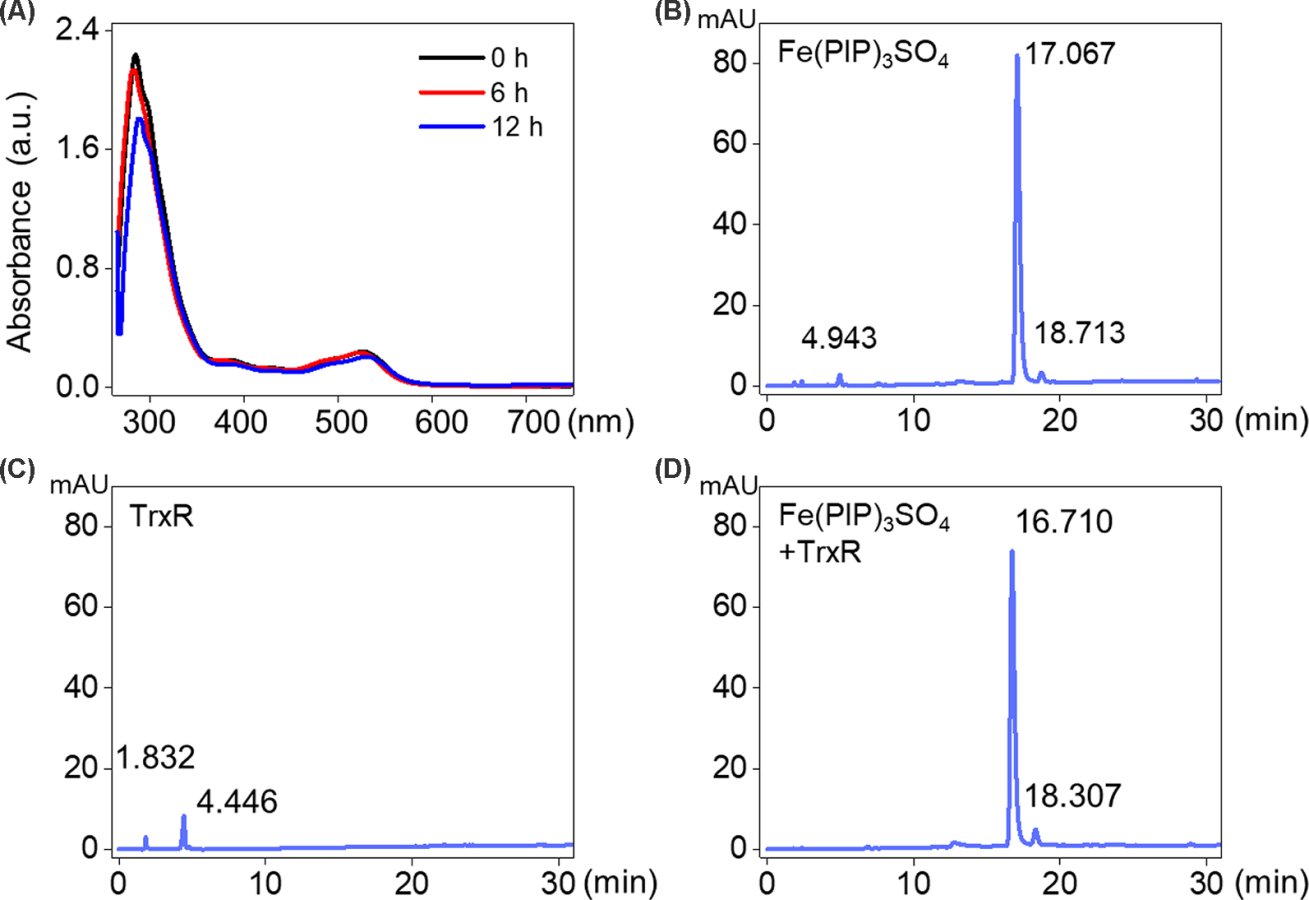
Interaction between Fe(PIP)_3_SO_4_ and TrxR. **(A)** UV-Vis spectrum of TrxR treated with Fe(PIP)_3_SO_4_ after incubation for 6 and 12 h. **(B)** HPLC analysis of Fe(PIP)_3_SO_4_ for 12 h. **(C)** HPLC analysis of TrxR for 12 h. **(D)** HPLC analysis of TrxR treated with Fe(PIP)_3_SO_4_ for 12 h.

**Figure 6:**
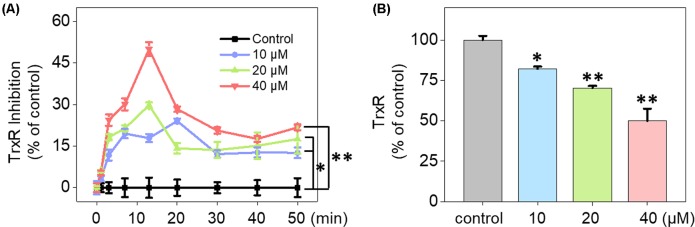
Inhibition of TrxR activity triggered by Fe(PIP)_3_SO_4_. **(A)** Inhibitory efficacy of cellular TrxR activity in U87 cells triggered by Fe(PIP)_3_SO_4_ in 50 min. **(B)** The most remarkable variation of cellular TrxR activity in U87 cells induced by Fe(PIP)_3_SO_4_ at 13-min time point. Values are presented as means ± SD in triplicate. Significant difference between the treatment and control groups is indicated at the *P*<0.05 (*) or *P*<0.01 (**) level.

UV-Vis spectra analysis was used to confirm the interaction between Fe(PIP)_3_SO_4_ and TrxR peptide. As shown in **Figure 5A**, substantial decrement could be noticed in absorbance at 297 nm for Fe(PIP)_3_SO_4_ after incubation with TrxR for 12 h. Furthermore, characteristic absorbance at 297 nm decreased further after 12-h incubation, with retention time of Fe(PIP)_3_SO_4_ (**Figure 5B**), the same as Fe atom attached to TrxR (**Figures 5C, D**). The characteristic absorption of Fe(PIP)_3_SO_4_ (intensity = 81.94 mAU) declined after 12-h exposure to TrxR (intensity = 80.97 mAU). Encouraged by these results, inhibition of TrxR activity by Fe(PIP)_3_SO_4_ (10, 20, and 40 μM, respectively) was tested by using the TrxR assay kit. As shown in **Figure 6A**, Fe(PIP)_3_SO_4_ markedly inhibited activity of TrxR, almost reaching 50% at 13 min, followed by declining to 20.1% at 50 min of incubation with Fe(PIP)_3_SO_4_ (40 μM). As shown in **Figure 6B**, in the initial stage, TrxR inhibition reached 50% at 13 min of incubation with Fe(PIP)_3_SO_4_ (40 μM). Simultaneously, relative TrxR activity declined to 78.2% and 69.5% triggered by Fe(PIP)_3_SO_4_ (10 and 20 μM, respectively).

### Permeability Across BBB by Fe(PIP)_3_SO_4_ Both *In Vitro* and *In Vivo*

**Figure 7:**
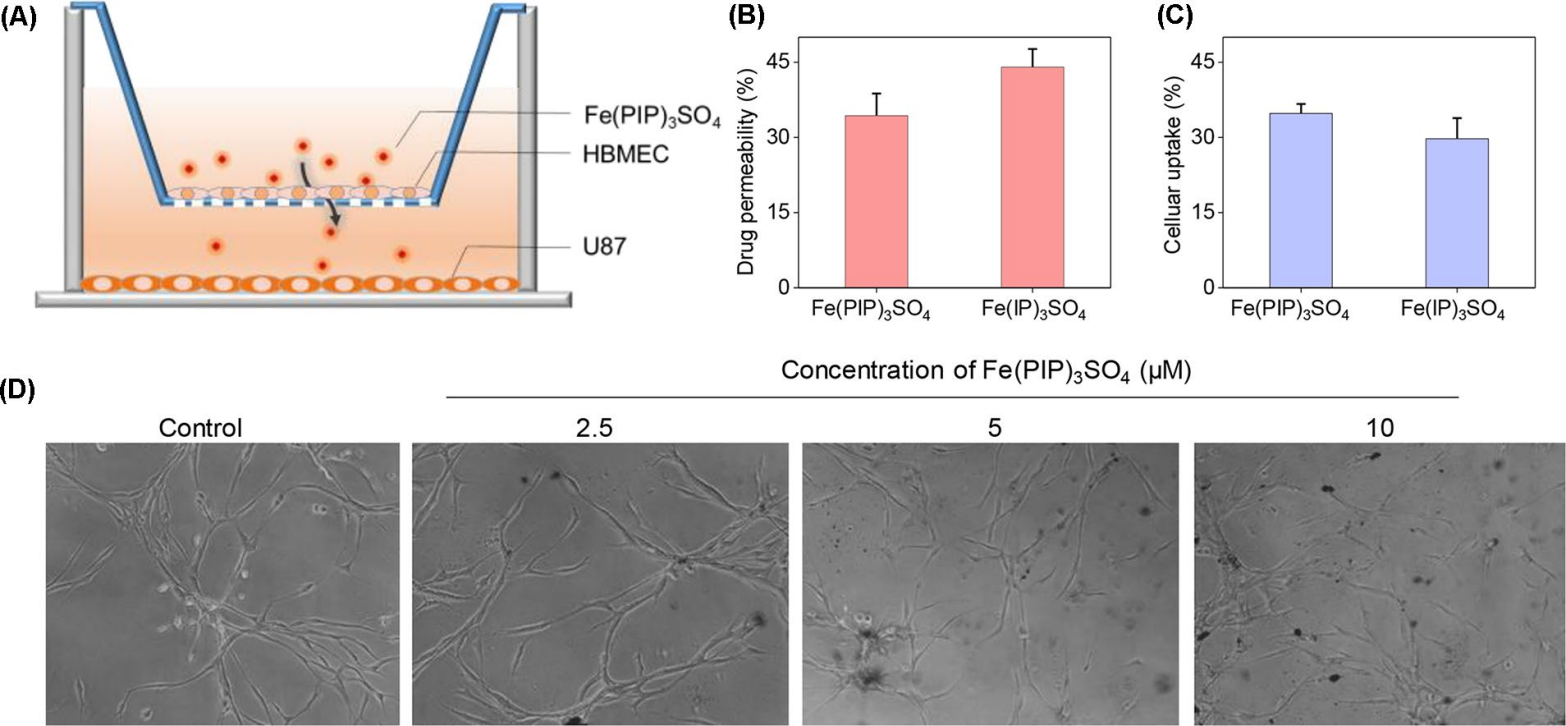
Permeability across BBB and inhibition of VM capability induced by Fe(PIP)_3_SO_4_. **(A)** Schematic diagram for coculture mimic BBB model. **(B)** Transmittance ratio of Fe(PIP)_3_SO_4_ and Fe(IP)_3_SO_4_ across BBB. **(C)** Cellular uptake of Fe(PIP)_3_SO_4_ on U87 cells after penetrating BBB for 24 h. Values are presented as means ± SD in triplicate. The treated concentration for Fe(PIP)_3_SO_4_ and Fe(IP)_3_SO_4_ were 10 μM. Values are presented as means ± SD in triplicate. **(D)** Inhibition of VM capability of U87 cells induced by Fe(PIP)_3_SO_4_ (2.5, 5, and 10 μM, respectively) for 24 h.

**Figure 8:**
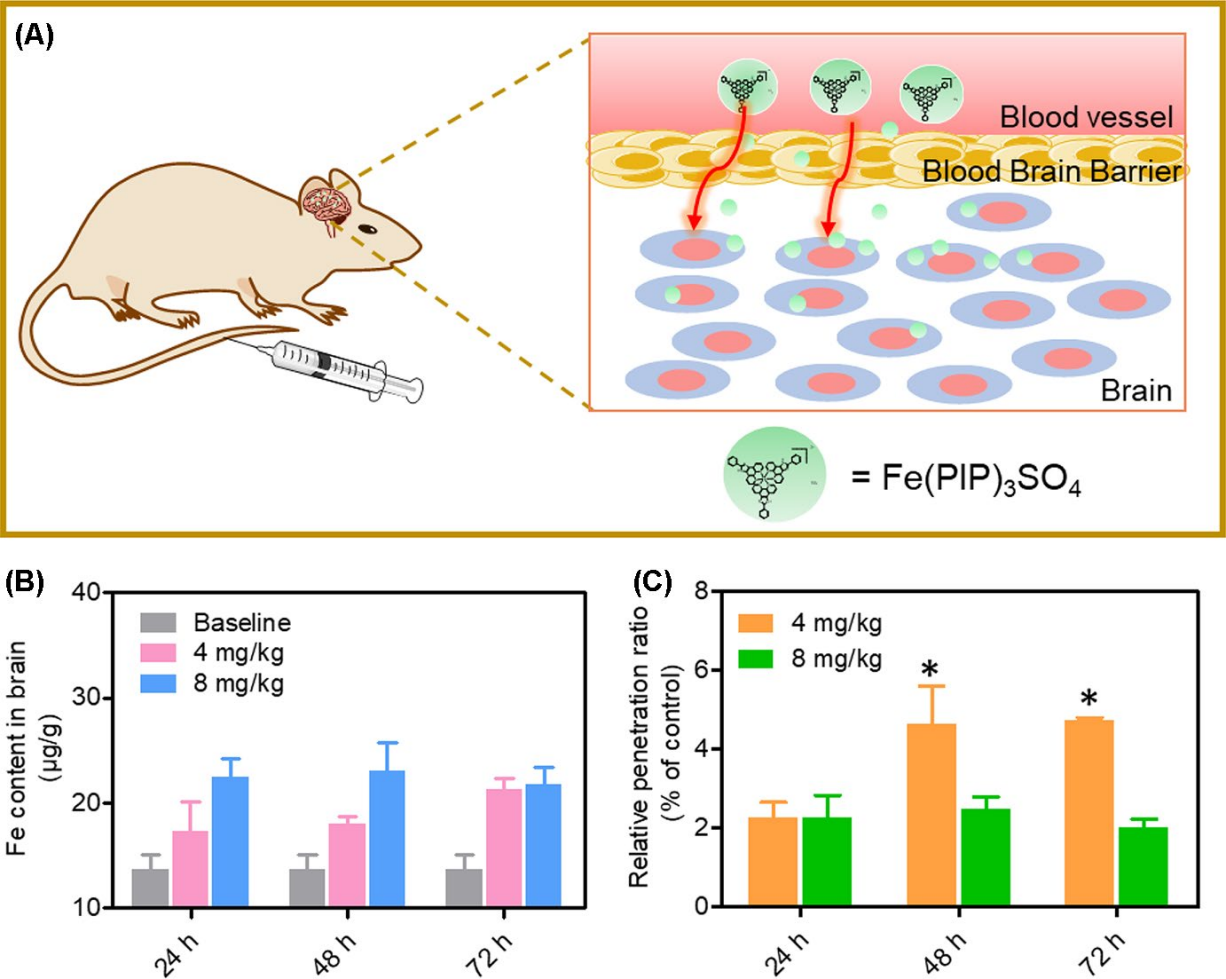
*In vivo* biodistribution of Fe content in the brains after administration (i.v.). **(A)** Schematic diagram for the experimental design. **(B)** *In vivo* Fe content in the brains after i.v. administration for 24, 48, and 72 h, respectively. The gray histograms were represented for the control group; the pink and blue histograms were represented for Fe content levels in brain treated with Fe(PIP)_3_SO_4_ (4 and 8 mg/kg, respectively). Values are presented as means ± SD in triplicate. Significant difference between treatment groups was indicated at the *P* < 0.05 (*) level. **(C)** Relative penetration ratio across BBB after i.v. administration for 24, 48 and 72 h, respectively. The yellow and green histograms were represented for the Fe content inside the brains treated with Fe(PIP)_3_SO_4_ (4 and 8 mg/kg, respectively). Values are presented as means ± SD in triplicate. Significant difference between treatment groups is indicated at the *P* < 0.05 (*) level.

A mimic BBB cell model containing the HBMEC/U87 coculture system was constructed *in vitro* (**Figure 7A**), and then the penetrating capability of Fe(PIP)_3_SO_4_ and Fe(IP)_3_SO_4_ (introduced as a comparing group) across BBB model was evaluated. **Figure 7B** showed that the penetrating percentage of Fe(PIP)_3_SO_4_ and Fe(IP)_3_SO_4_ was 34.6% and 44.3%, respectively. Uptake amount of the complex toward U87 cells in the lower compartment was also tested. **Figure 7C** showed cellular uptake percentage of Fe(PIP)_3_SO_4_ was 32.4%, which was relatively higher than that of Fe(IP)_3_SO_4_ (29.5%). As it is imperative to improve effective antiglioblastoma agents, simultaneously inhibiting the VM capability of cancer cells, we then examined the destruction of VM channels after incubation with Fe(PIP)_3_SO_4_ (2.5, 5, and 10 μM) for 24 h. Results showed that U87 cells formed VM channels (control group), and serious inhibition of VM channels was noticed by Fe(PIP)_3_SO_4_ in a dose-dependent manner. For instance, inhibition level induced by Fe(PIP)_3_SO_4_ (10 μM) greatly increased compared with control (**Figure 7D**). Additionally, Fe(PIP)_3_SO_4_ (20 μM) exhibited slightly weaker permeability into U87 tumor spheroids. Based on the findings above, *in vivo* biodistribution of Fe content in the brains was tested on mice by ICP-OES analysis (**Figure 8A**); results showed that Fe content in the brains were 17.26, 18.03, and 21.35 μg/g (dosage of 4 mg/kg for 24, 48, and 72 h, respectively), which showed moderately increased penetrating behavior. In addition, Fe contents in the brains were 22.48, 23.10, and 21.81 μg/g (dosage of 8 mg/kg for 24, 48, and 72 h, respectively), with no obvious changes observed (**Figure 8B**). One more to mention, the relative penetration rates of Fe(PIP)_3_SO_4_ (4 mg/kg) were 2.25%, 4.65%, and 4.73%, respectively (after i.v. administration for 24, 48, and 72 h), and the dose of 8 mg/kg led to the relative penetration rate increases to 2.26%, 2.48% and 2.02%, respectively (after i.v. administration for 24, 48, and 72 h) (**Figure 8C**).

### Inhibitory Effects and Penetrating Ability of Fe(PIP)_3_SO_4_ on U87 Tumor Spheroids

**Figure 9:**
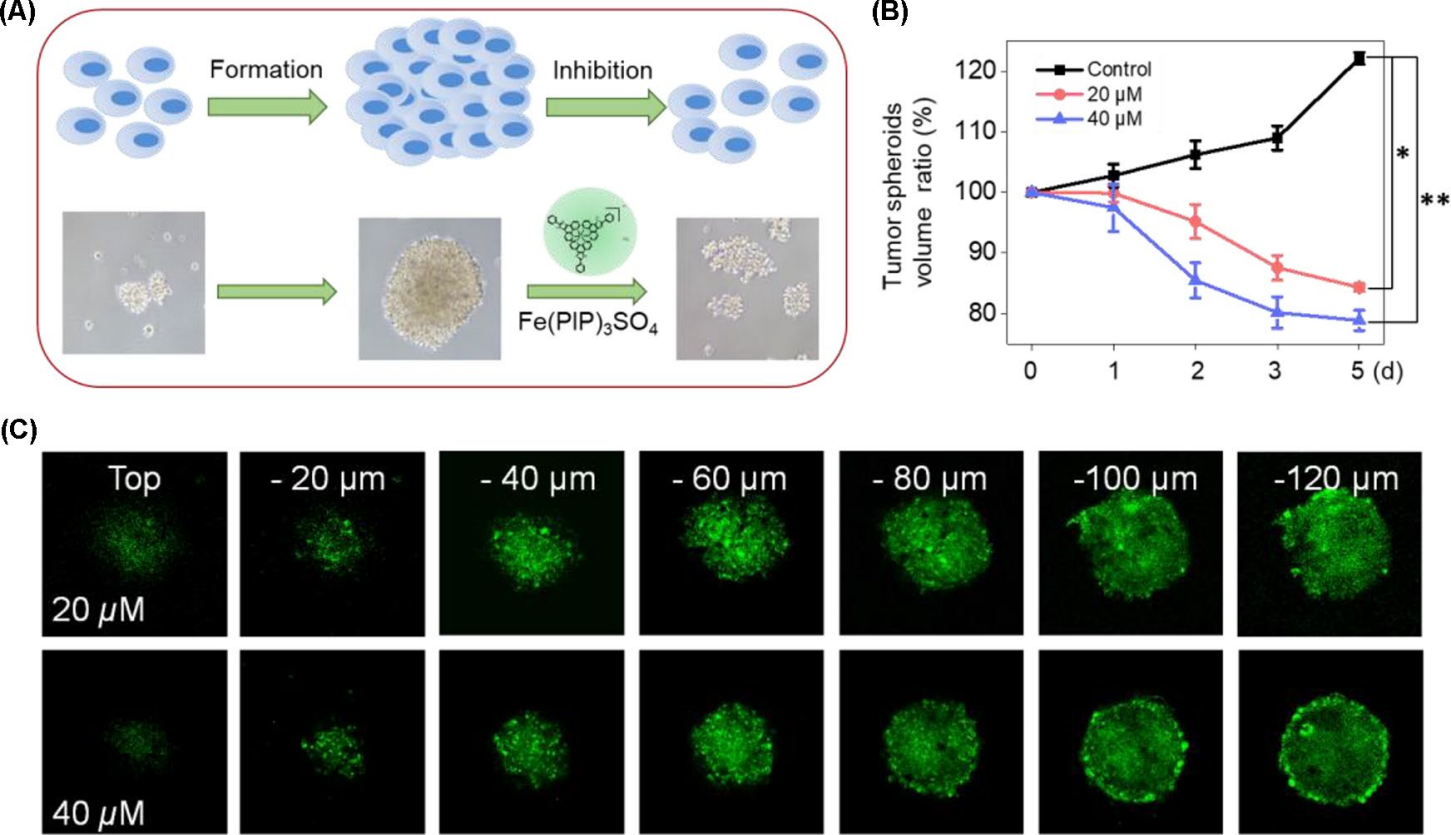
Penetrating capability and inhibitory effects of Fe(PIP)_3_SO_4_ on U87 tumor spheroids. **(A)** Schematic diagram for the experimental design. **(B)** Inhibitory effects of U87 tumor spheroids treated with Fe(PIP)_3_SO_4_ (20 and 40 μM, respectively) for consecutive days. Significant difference between the treated and control groups is indicated at the *P* < 0.05 (*) or *P* < 0.01 (**) level. **(C)** Penetrating ability for Fe(PIP)_3_SO_4_ (20 and 40 μM, respectively) into the brain tumor spheroids for 12 h.

U87 tumor spheroids were successfully formed, with diameters up to 200 μm for each spheroid after careful cultivation for 5 to 7 days (**Figure 9A**). Then, the growth kinetics of tumor spheroids and inhibitory effects of Fe(PIP)_3_SO_4_ (20 and 40 μM, respectively) were evaluated on several consecutive days. Results showed that U87 tumor spheroids bulk decreased when treated with Fe(PIP)_3_SO_4_ (20 and 40 μM, respectively), compared with the sustainable growth of the control group. After incubation with Fe(PIP)_3_SO_4_ (20 and 40 μM, respectively) for 5 days, relative tumor spheroid volume declined to 87.4% and 76.8% (compared with 100% control group). In addition, relative tumor spheroid volume of control raised up to 123.8% approximately, which further demonstrated favorable inhibitory efficacy of Fe(PIP)_3_SO_4_ against tumor spheroids (**Figures 9A, B**). Then, the entry of drug into the tumor spheroids was visualized on a confocal laser-scanning microscope. First, tumor spheroids were cultured with diameters up to 200 μm, which were capable of simulating the pathological features of some solid tumors. Afterward, fluorescence images were obtained every 20 μm of the spheroid layers, from top to the middle zone (at −120 μm), after incubation with Fe(PIP)_3_SO_4_ (20 and 40 μM) for 12 h. And the tumor spheroids treated with Fe(PIP)_3_SO_4_ (40 μM) displayed stronger fluorescent intensity even in the deep layer of −120 μm (**Figure 9C**).

### *In Vivo* Toxicity and Biodistribution of Fe(PIP)_3_SO_4_

**Figure 10:**
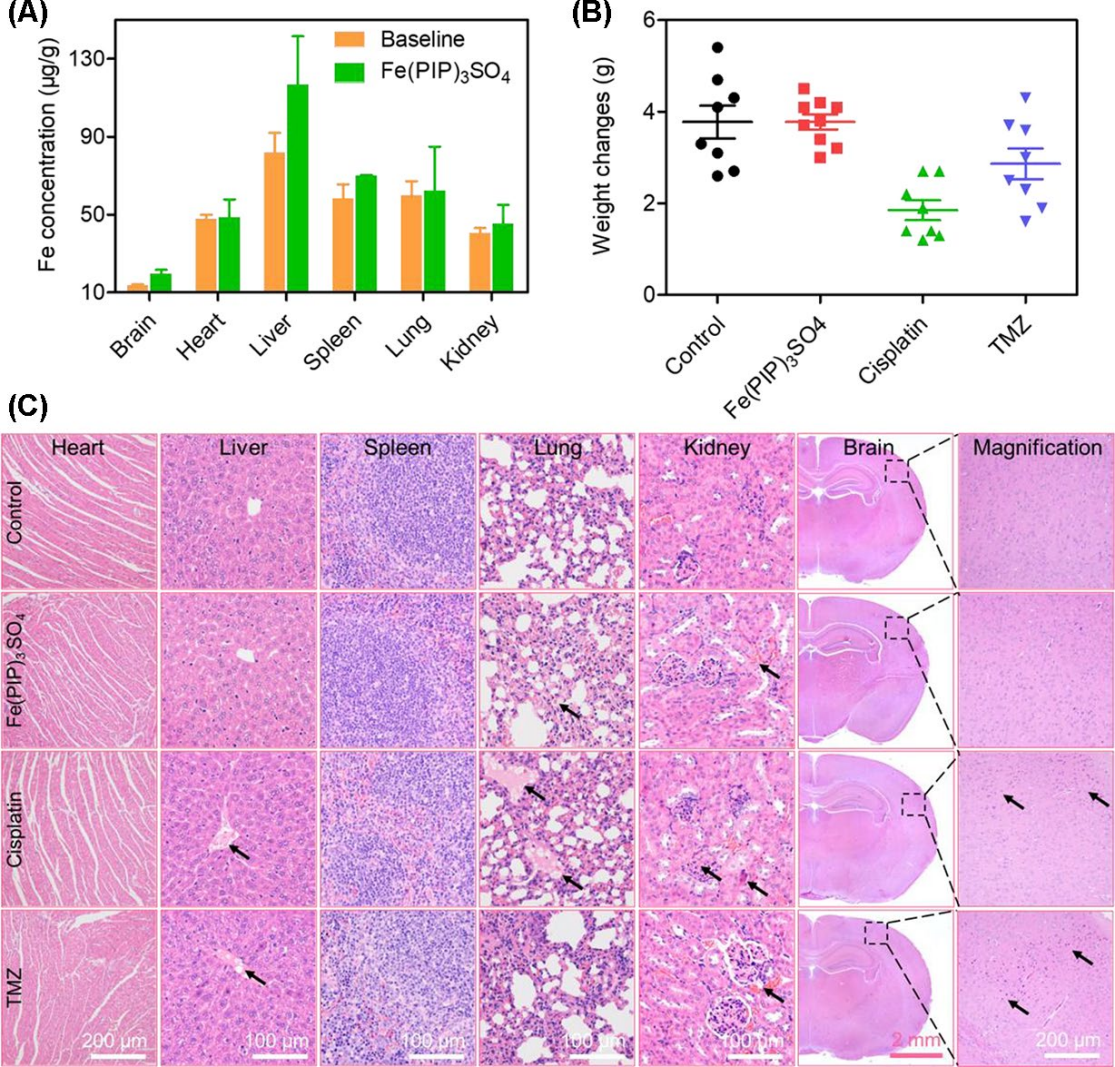
*In vivo* toxicity and biodistribution of Fe(PIP)_3_SO_4_ after 72 h. **(A)** *In vivo* biodistribution of Fe(PIP)_3_SO_4_ in major organs after i.v. administration for 72 h. The yellow and green histograms represent the baseline and Fe(PIP)_3_SO_4_ groups, respectively. Values are presented as means ± SD in triplicate. **(B)** Body weight growth treated with Fe(PIP)_3_SO_4_, cisplatin, and TMZ after 72 h. **(C)** H&E staining of heart, liver, spleen, lung, kidney, and brain in NIH mice after treatments with Fe(PIP)_3_SO_4_, cisplatin, and TMZ for 72 h.

**Figure 11:**
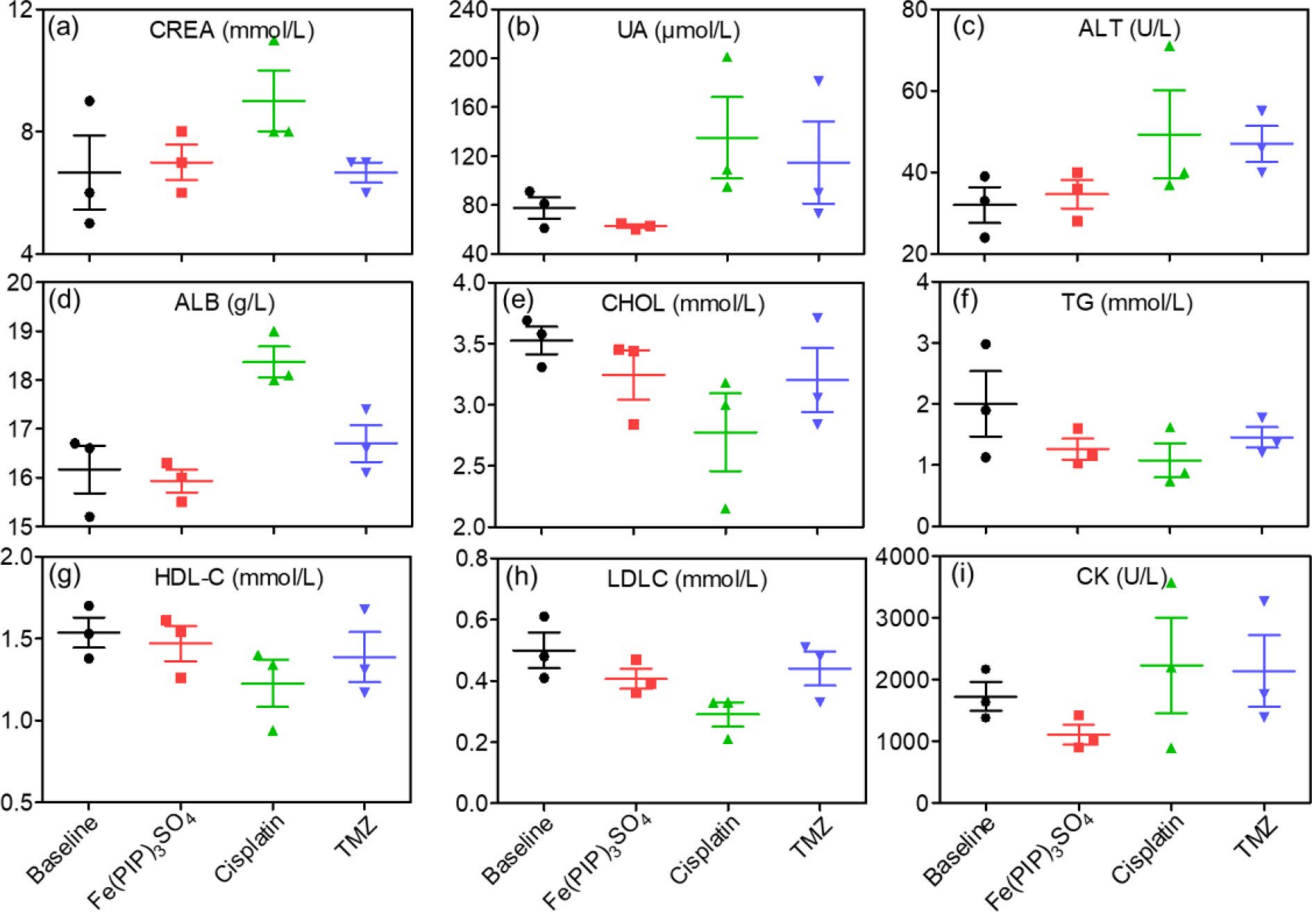
Hematological analysis of NIH mice after treatment with Fe(PIP)_3_SO_4_, cisplatin, and TMZ for 72 h. **(A)** CREA indicates creatinine. **(B)** UA indicates uric acid. **(C)** ALT indicates alanine transaminase. **(D)** ALB indicates serum albumin. **(E)** CHOL indicates cholesterol. **(F)** TG indicates triglyceride. **(G)** HDL-C indicates high-density lipoprotein cholesterol. **(H)** CK indicates CK. **(I)** LDLC indicates low-density lipoprotein cholesterol.

Biodistribution and toxicity of Fe(PIP)_3_SO_4_ were acquired by using the NIH mice treated with Fe(PIP)_3_SO_4_, cisplatin, and TMZ (4 mg/kg) for 72 h. The results of quantitative determination of Fe content manifested that Fe content increased by 1.43-fold in the brains compared with control (19.64 and 13.71 μg/g, respectively). Fe content could also be noticed in the livers; the drug accumulation of Fe increased by 1.43-fold compared with the control group (116.68 and 81.79 μg/g, respectively). After treatment with different drugs, the weight growth of mice seemed lower in the cisplatin- and TMZ-treated groups (1.8 and 2.8 g, respectively), whereas no obvious weight growth could be noted in the Fe(PIP)_3_SO_4_ group compared with control (3.82 and 3.85 g, respectively) (**Figures 10A, B**). Furthermore, H&E staining was utilized to study the toxic effects of Fe(PIP)_3_SO_4_, cisplatin, and TMZ on major organs in mice. As shown in **Figure 10C**, bleeding and fatty degeneration could be noticed in liver cells treated with cisplatin and TMZ (4 mg/kg) (indicated by the arrows). In contrast, Fe(PIP)_3_SO_4_ caused negligible damage on normal organs, including slight bleeding and fatty degeneration in pulmonary alveoli and kidney cells. Additionally, fatty degeneration could be noted in pulmonary alveoli treated with cisplatin and TMZ; overt apoptosis and vesicular degeneration could also be detected in liver cells triggered by cisplatin and TMZ (indicated by the arrows). More than that, the number of intact neurons in cortical regions was slightly decreased after treatment with Fe(PIP)_3_SO_4_. In comparison, obvious loss of intact neurons and irregular morphology could be noticed in cortical regions induced by cisplatin and TMZ (indicated by the arrows in the magnified zone of brain tissue). Hematological analysis results showed that Fe(PIP)_3_SO_4_ caused slight changes on cholesterol (CHOL), triglyceride (TG), low-density lipoprotein cholesterol (LDLC), and creatine kinase (CK) index, with no obvious changes shown in creatinine (CREA), uric acid (UA), alanine transaminase (ALT), albumin (ALB), and high-density lipoprotein cholesterol (HDL-C) index, which caused less hematological changes than cisplatin and TMZ. For instance, Fe(PIP)_3_SO_4_ could induce UA index to 63 μmol/L, compared with 77 μmol/L (control group), whereas cisplatin and TMZ changed UA index to 135 and 115 μmol/L, respectively (**Figures 11A-I**).

## Discussion

Glioblastoma has developed as a hard issue to tackle with for decades, considering that a small portion of chemotherapeutic drugs could efficiently cross the BBB into the brain (Utsuki et al., 2006; Neuwelt et al., 2011). Besides, VM channels have been an obstacle for clinical treatments for the glioblastoma (Sharma et al., 2018). Hence, it is urgently required to develop promising candidates to efficiently permeate the BBB, simultaneously disrupting the VM capability (Chen and Chen, 2014). On the other hand, Fe (II) complex exhibits favorable anticancer efficacy by binding with intracellular DNA and regulating ROS levels. Based on our previous studies, a series of Fe (II) polypyridyl complexes have been synthesized and studied as potential anticancer agents by interrupting TrxR system and regulating cell cycle (Chen et al., 2015). More than that, further developments of Fe (II) complex–based anticancer agents accompanied with tumor necrosis factor–related apoptosis‐inducing ligand for the treatment of glioblastoma were demonstrated as reported by Lin et al. (2018). Translation initiation factor 4E-BP1 could selectively stop the translation of mRNA, which is necessary for cell proliferation by inducing cycle arrest, thus promoting drug resistance of glioblastoma (Zhu et al., 2014). Clinical data have confirmed that highly phosphorylated 4E-BP1 is associated with poor prognosis and in certain tumors, such as glioblastomas (Rojo et al., 2007).

Previously, Fe (II) complexes with lipophilicity were synthesized in our laboratory through a facile method, of which the anticancer activity was determined to be effective (Chen et al., 2015). Hence, the aim of this study was to determine anticancer activity of Fe (II) polypyridyl complex, Fe(PIP)_3_SO_4_ (PIP = 2-phenylimizazo[4,5-*f*] [1,10] phenanthroline) (Chen et al., 2015) against U87 malignant cancer cells. 3-(4,5-Dimethyl-2-thiazolyl)-2,5-diphenyl-2-H-tetrazolium bromide (MTT) assay was conducted to study the cytotoxic effects of Fe(PIP)_3_SO_4_ toward glioma cells and the side effects on HEB (normal human glial cells), and half inhibitory concentration (IC_50_) values were introduced to study cytotoxicity. Results show that Fe(PIP)_3_SO_4_ exhibits stronger anticancer efficacy toward U87 cells than TMZ. It is interesting that the cytotoxicity of Fe(PIP)_3_SO_4_ toward HEB cells (human normal glial cells) was lower than that of U87 cells (human malignant glioma cells), indicating its safety potency in future application. The safety index (SI) of Fe(PIP)_3_SO_4_ was higher than that of cisplatin (SI 2.7 vs. 1.4), also showing potential application value (Chen et al., 2015) . As U87 malignant glioma cells are regarded as one of the malignant human tumor cells, the cell lines are used for the following study of anticancer mechanisms of Fe(PIP)_3_SO_4_. Furthermore, we found that internalization content of Fe(PIP)_3_SO_4_ in U87 cells was much higher than that of HEB cells (**Figure 1B**), which might be related with the lipophilicity and planarity of Fe(PIP)_3_SO_4_ (Luo et al., 2014).

Apoptosis and cycle arrest are considered as 2 major mechanisms to inhibit cell proliferation (Li et al., 2011; Pierroz et al., 2012; Cao et al., 2013). Moreover, previous studies have shown that Fe (II) complexes containing phenanthroline could regulate proliferation of cancer cells by cell cycle arrest (Chen et al., 2015). Results in **Figure 2** suggest that cell death in U87 cells induced by Fe(PIP)_3_SO_4_ was triggered by G2/M cell cycle arrest and early apoptosis during the early stage of 24-h incubation and apoptosis (especially for late apoptosis) gradually as exposure time extended to 72 h. In addition, Annexin V/PI-flow cytometric analysis indicates that lower dose of Fe(PIP)_3_SO_4_ (concentrations of 5 and 10 μM) significantly increased the amount of early apoptotic cells (Annexin V–positive/PI-negative cells). In addition, higher dose of Fe(PIP)_3_SO_4_ (concentrations of 20 and 40 μM) significantly increased the number of late apoptotic cells (Annexin V–negative/PI-positive cells). To further study anticancer mechanism of Fe(PIP)_3_SO_4_ to U87 cells, Western blotting assay was conducted to study related signals. 4E-BP1 is a downstream effector molecule of the classical mTOR pathway, belonging to the family member of translation repressor proteins (Dodd et al., 2015). More than that, 4E-BP1 inhibits the translation of 5′ cap-dependent proteins and hinders protein synthesis (Gardner et al., 2015). In addition, 4E-BP1 can selectively inhibit the translation of mRNA necessary for cell proliferation to trigger cell cycle arrest, such as ornithine decarboxylase and cyclin D1 (Faller et al., 2015). As shown in **Figure 2C**, Fe(PIP)_3_SO_4_ suppressed the expression of signaling proteins, including cyclin B1 and p4E-BP1, stopping G2/M phase cell cycle arrest by restricting the initiation process of protein translation. Furthermore, the expression level of p53 signal (Halaby et al., 2015) was also investigated after incubation with Fe(PIP)_3_SO_4_. Besides, phosphorylation level of p53 at Ser 15 could trigger cycle arrest and early cell apoptosis caused by chemotherapeutic and chemopreventive Fe complexes (Reshi et al., 2016). Taken together, Fe(PIP)_3_SO_4_ could induce G2/M phase cycle retardation and apoptosis (early apoptosis followed by late apoptosis in a dose-dependent manner) by regulating p53 and 4E-BP1 expression.

Imbalance of ROS is considered as a principal way to inhibit cell proliferation process (Gandin et al., 2010; Gorrini et al., 2013). Results in **Figure 4** demonstrate that Fe(PIP)_3_SO_4_ could downregulate ROS levels in U87 cells, thanks to the favorable free radical–scavenging capability (Gao et al., 2013). More than that, TrxR is a major protein in the regulation of the redox equilibrium system in cells (Liu et al., 2013). Phenanthroline Ru(II) compounds could target TrxR and induce excessive ROS production, which was related with cellular DNA damage and cellular apoptosis (Zeng et al., 2016). One more to mention, Fe (II) and gold complexes are capable of targeting TrxR and trigger intracellular ROS generation (Jurgens et al., 2017). The findings for binding effect of metal complex with TrxR encourage us to further study anti-caner activity of Fe(PIP)3SO4.

The BBB model, which separates the brain and the peripheral blood circulation system, has been considered as a main obstacle to clinical treatments of malignant gliomas, especially hindering the entry of some drugs into the cerebral circulation (Gao et al., 2014). As is known, glioblastoma multiforme is regarded as one of the most malignant brain tumors (Zhan et al., 2012). Therefore, the capability of promising candidates to penetrate across BBB is a key element for efficient treatment of gliomas. To ascertain the capacity of Fe(PIP)_3_SO_4_ to permeate across BBB, a HBMEC/U87 coculture structure system as a mimic BBB model was established according to a previous report (Mo et al., 2016). Results in **Figure 7** suggest that Fe (II) complex transmitted across BBB; thus, Fe(PIP)_3_SO_4_ was promising as a metal-based antiglioblastoma agent. Additionally, VM ability of glioma cells is considered as one of the major barriers to ameliorating the invasion and/or drug resistance of gliomas (Zhang et al., 2007). Hence, advancing effective antiglioblastoma agents simultaneously inhibiting VM ability of cancer cells should be at the top priority of researchers’ agenda. Results demonstrate that VM channels were markedly suppressed after incubation with Fe(PIP)_3_SO_4_ for 24 h, exhibiting vigorous permeability through BBB and inhibiting VM channels as a potential metal-based antiglioma agent. Nevertheless, more than 98% of small molecules are compromised by compact structures of endothelial cells of BBB to efficiently penetrate into the brains (Madsen and Hischberg, 2010). For instance, approximately 9% to 10% of TMZ could accumulate in the brains after 0.5 h of administration, followed by descending to only 1% after 24 h of administration, showing very low bioavailability (Khan et al., 2016; Prabhu et al., 2017). *In vivo* biodistribution of Fe in the brains after i.v. administration with Fe(PIP)_3_SO_4_ was also determined. Results in **Figure 8** show that Fe content accumulated in the brain in a time-dependent manner. For example, at 72-h time point, 23 μg/g Fe was detected in the brains, indicating that Fe(PIP)_3_SO_4_ could penetrate across BBB efficiently *in vivo*, while Fe content in the brain remained steady when treated with 8 mg/kg of Fe(PIP)_3_SO_4_. Furthermore, the penetration rate of Fe(PIP)_3_SO_4_ (4mg/kg) increased from 2.25% to 4.73% in a time-dependent manner, which was higher than that of TMZ as previously described (Prabhu et al., 2017). Results above demonstrate that Fe(PIP)_3_SO_4_ exhibits relatively potent penetrating ability across BBB and could be advanced for further antitumor studies on animal levels in the future. These findings were further confirmed by tumor spheroid model assay. Studies have reported that tumor spheroids with diameters of about 200 to 300 μm are capable of simulating the pathological features of some solid tumors, such as the distinctive hypoxic areas in the central area (You et al., 2016). Tumor spheroid simulating models could be exploited to simulate the tumor tissue *in vitro*. It was suggested that Fe(PIP)_3_SO_4_ could penetrate through BBB and accumulate in the interior of the brains. Moreover, *in vivo* toxicity study was conducted for safety evaluation. Results in **Figures 10** and **11** show that Fe(PIP)_3_SO_4_ exhibits relatively lower toxicity on normal tissues (such as the livers, lungs, and brains compared with cisplatin and TMZ). It is interesting that Fe(PIP)_3_SO_4_ seemed lower than that of the cytotoxicity results mentioned above, both compared with TMZ, which demonstrates that Fe(PIP)_3_SO_4_ is of low toxicity as a promising anticancer candidate.

## Conclusion

Herein, we evaluated the anticancer activity of Fe(PIP)_3_SO_4_ *in vitro* and acute toxicity and biodistribution *in vivo*. Results above demonstrate that Fe(PIP)_3_SO_4_ induced G2/M phase retardation and apoptosis (early apoptosis followed by late apoptosis in a dose-dependent manner) by regulating p53 and 4E-BP1 expression. In addition, this Fe (II) complex inhibited cell proliferation by suppressing intracellular ROS levels by well free radical–scavenging capability and interaction with TrxR. Furthermore, lipophilic Fe(PIP)_3_SO_4_ could penetrate across BBB both *in vitro and in vivo* and could simultaneously destroy VM channels, which indicated potential antiglioblastoma efficacy. In summary, Fe(PIP)_3_SO_4_, an Fe (II)-based polypyridine complex, exhibited favorable anticancer activities against gliomas by regulating expression level of 4E-BP1 and p-53, making it a promising and low-toxicity chemotherapeutic agent to antagonize glioblastoma and overcome BBB.

## Ethics Statement

This animal study was carried out in accordance with the principles of the Basel Declaration and recommendations of the Institutional Animal Use and Care regulations of Jinan University.

## Author Contributions

CD and HZ contributed to the design and execution of the experimental studies, analysis and interpretation of the results, and draft and writing of the manuscript. LH contributed to the execution of the experimental studies. AX and TC contributed to the research design and critical review.

## Funding

This work was supported by the Natural Science Foundation of China (21877049), Major Program for Tackling Key Problems of Industrial Technology in Guangzhou (201902020013), Dedicated Fund for Promoting High-Quality Marine Economic Development in Guangdong Province (GDOE-2019-A31), the Natural Science Foundation of Guangdong Province (no. 2016A030313109, 2015A030310019), and the Scientific Research and Fostering Foundation of Jinan University (no. 2016315, 2015206).

## Conflict of Interest Statement

The authors declare that the research was conducted in the absence of any commercial or financial relationships that could be construed as a potential conflict of interest.
