# Supplementary material for: Iron (II) Polypyridyl Complexes as Antiglioblastoma Agents to Overcome the Blood-Brain Barrier and Inhibit Cell Proliferation by Regulating p53 and 4E-BP1 Pathways
Source: Front Pharmacol. 2019 Sep 3;10:946. doi: 10.3389/fphar.2019.00946 (PMC6733960; doi:10.3389/fphar.2019.00946)
Supplement: Supplementary file 1 [file Table_1.docx]

***Supplementary Material***

**Iron (II) polypyridyl complexes as anti-glioblastoma agents to overcome the blood-brain barrier and induce G2/M phase cell cycle arrest by regulating p53 and 4E-BP1 pathways**

**Huili Zhu ^b#^, Chengli Dai ^a#^, Lizhen He ^a^, Anding Xu ^b^*, Tianfeng Chen ^a^***

^a^ Department of chemistry, Jinan University, Guangzhou 510632, China

^b^ Department of Neurology and Stroke Center, The first Affiliated Hospital, Jinan University, Guangzhou 510632, Guangdong, China

*Corresponding author. Department of chemistry, Jinan University, Guangzhou 510632, China.

Department of Neurology and Stroke Center, The first Affiliated Hospital, Jinan University, Guangzhou 510632, Guangdong, China

Fax: (+): 86-20-85220223, E-mail: [tchentf@jnu.edu.cn](mailto:tchentf@jnu.edu.cn). [tlil@jnu.edu.cn](mailto:tlil@jnu.edu.cn)

**Table S1.** Table of IC_50_ values of Fe(PIP)_3_SO_4_ towards glioma and normal cell lines

| Compounds |  |  | IC_50_^a^ (μM) |  |  | SI^b^ |
| --- | --- | --- | --- | --- | --- | --- |
|  | U87 | U251 | C6 | HEB | CHEM5 |  |
| Fe(PIP)_3_SO_4_ | 9.35 | 4.44 | 3.54 | 36.03 | 4.68 | 3.85 |
| TMZ | 92.87 | 24.49 | 82.39 | 593.6 | 289.55 | 6.39 |

a: IC_50_ is represented for 50% inhibitory concentration (μM).

b: Safety index (SI) = IC_50_ _(HEB cells)_/ IC_50 (U87 cells)_.

IC_50_ values of Fe(PIP)_3_SO_4_ towards gliomas (U87, U251 and C6 cells) and normal cell lines (HEB and CHEM5 cells) after incubation for 72h. Cells with blue markers represented for cancer cell lines, and cells with red markers represented for normal cell lines. Values expressed were means ± SD of triplicate.

**Figure S1**. Quantitative analysis of sub-G1 phase and G2/M phase accumulation effected by Fe(PIP)_3_SO_4_ (5, 10, 20 and 40 μM, respectively) for 36 h and sub-G1 phase & G2/M phase accumulation induced by Fe(PIP)_3_SO_4_ (20 μM) for different time on retardation and apoptotic cell death in U87 cells. Values were represented as means ± SD of triplicate. Significant difference between treatment and control group is indicated at *P*＜0.05 (*) or *P*＜0.01 (**) levels.
